# DsbA-L activates TGF-β1/SMAD3 signaling and M2 macrophage polarization by stimulating AKT1 and NLRP3 to promote pulmonary fibrosis

**DOI:** 10.1186/s10020-024-00983-9

**Published:** 2024-11-23

**Authors:** Juan Wang, Zhenkun Xia, Bei Qing, Ying Chen, Linguo Gu, Hongzuo Chen, Zhenglian Ge, Yunchang Yuan

**Affiliations:** Department of Thoracic Surgery, The Second Xiangya Hospital of Central South University, Changsha, Hunan China

**Keywords:** Pulmonary fibrosis, DsbA-L, TGF-β1/SMAD3 signaling, M2 macrophage polarization, AKT1, NLRP3

## Abstract

**Background:**

Pulmonary fibrosis (PF) is a progressive and difficult-to-heal lung disease that poses a significant threat to human life and health. This study aimed to investigate the potential pathological mechanisms of PF and to identify new avenues for the treatment of PF.

**Methods:**

Clinical samples were collected to assess the effect of disulfide-bond A oxidoreductase-like protein (DsbA-L) on PF. TGF-β1-induced MLE-12 cell model and bleomycin (BLM)-induced mice model were established. Changes in physiological morphology and fibrosis were observed in the lung tissues. The degree of apoptosis and the mitochondrial function was analyzed. The expression of relative cytokines was examined. The CD68^+^/CD206^+^ ratio was determined to indicate M2 macrophage polarization.

**Results:**

The expression of DsbA-L was upregulated in patients with PF and PF-like models. In vitro, DsbA-L overexpression exacerbated TGF-β1-induced the deposition of extracellular matrix (ECM), apoptosis, inflammation, and mitochondrial damage, whereas DsbA-L silencing exerted the opposite effects. DsbA-L silencing inhibited the activation of AKT1, NLRP3, and SMAD3 by TGF-β1. MLE-12 cells silencing DsbA-L limited the polarization of RAW264.7 cells towards the M2 phenotype. AKT1 agonist or NLRP3 agonist reversed the role of DsbA-L silencing in inhibiting the TGF-β1/SMAD3 pathway and M2 macrophage polarization. In vivo, *DsbA-L* knockout protected mice from PF-like pathological damage caused by BLM.

**Conclusion:**

DsbA-L exhibited a significant profibrotic effect in lung epithelial cells and mice, which increased the levels of AKT1 and NLRP3 to activate the TGF-β1/SMAD3 pathway and M2 macrophage polarization. These findings could shed light on new clues for comprehension and treatment of PF.

**Supplementary Information:**

The online version contains supplementary material available at 10.1186/s10020-024-00983-9.

## Introduction

Pulmonary fibrosis (PF), as a type of interstitial lung disease, is mainly characterized by injury to and aberrant repair of the alveolar epithelium, leading to structural destruction of lung tissue (Hewlett et al. 2018). The pathological changes of PF are mainly manifested by the aggregated proliferation of fibroblasts/myofibroblasts, excessive deposition of extracellular matrix (ECM) and diffuse inflammatory response (Lv et al. 2020). Idiopathic pulmonary fibrosis (IPF) is the most common under clinically diagnosed form of PF, and IPF is a progressive and difficult-to-treat disease with high mortality (Spagnolo et al. 2021). As the aging of the population continues to accelerate, the threat of IPF to human life, health and socioeconomics is expected to increase continuously. Therefore, it is an important research direction for researchers to explore the potential pathogenesis of PF and effective treatment.

Transforming growth factor-β1 (TGF-β1) is a key regulator of tissue fibrosis, which mainly induces the development of tissue fibrosis through the activation of small mother against decapentaplegic (SMAD) signaling (Hu et al. 2018). The expression levels of TGF-β and p-SMAD3 are significantly upregulated in the bleomycin (BLM)-induced PF animal model, while blocking TGF-β1/SMAD3 signaling effectively attenuates the progression of PF in mice (Guo et al. 2019). Accumulating evidence supports the role of macrophages in the pathogenesis of PF (Byrne et al. 2016; Ogawa et al. 2021). Macrophages are integral players in the host defense mechanisms that can polarize into two phenotypes based on the different microenvironments, including the pro-inflammatory phenotype (M1) and the anti-inflammatory phenotype (M2) (Smigiel and Parks 2018). M2 macrophages secrete copious amounts of TGF-β1 to promote fibroblast differentiation and proliferation, thereby exacerbating the development of PF (Wang, et al. 2021). Conversely, TGF-β1 induces macrophage polarization towards the M2 phenotype (Liu et al. 2019). These findings report the involvement of the TGF-β1/SMAD3 pathway and macrophages during PF.

Protein kinase B (PKB/AKT) is a serine/threonine protein kinase that stimulates the development of PF in response to upstream phosphatidylinositol-3 kinase (PI3K) signaling (Wang et al. 2022). Previous studies have demonstrated that the expression of AKT1 is elevated in IPF patients and that a reduction in the production of AKT1 significantly reduces the secretion of α-smooth-muscle actin (α-SAM) and fibronectin, as well as inhibiting the progression of TGF-β-induced PF (Nie et al. 2019; Abdalla et al. 2015). Furthermore, the NOD-like receptor protein 3 (NLRP3) inflammasome and its downstream cytokine caspase-1 are markedly activated in IPF mice, which are involved in epithelial-mesenchymal transition (EMT) via the TGF-β1 pathway (Tian et al. 2017).

Disulfide-bond A oxidoreductase-like protein (DsbA-L) was originally identified from rat liver mitochondria and has been shown to activate SMAD3 and NLRP3 signaling, which in turn exacerbates tubulointerstitial fibrosis and inflammation (Yang et al. 2021; Li et al. 2020). Nevertheless, the majority of research investigating the potential of DsbA-L in the treatment of fibrosis has focused on kidney tissue, with the role of this protein in PF remaining to be further elucidated. It is currently unclear whether DsbA-L influences TGF-β1/SMAD3 signaling and macrophage polarization during the development of PF. Therefore, this study aims to explore whether DsbA-L is involved in the development of PF and its potential molecular mechanism through in vitro and in vivo experiments to find different approaches for the treatment of PF.

## Methods

### Clinical samples

Lung tissues were obtained from non-PF and PF patients (n = 8) at the Second Xiangya Hospital of Central South University with informed consent. The diagnosis of PF was based on the American Thoracic Society (ATS)/European Respiratory Society (ERS) consensus diagnostic criteria. The human study was approved by the Ethics Committee of The Second Xiangya Hospital of Central South University (Approved protocol number: 2013001). Prior to their inclusion in the study, all participants were recruited with written informed consent.

### Bleomycin (BLM)-induced PF in mice

The specific DsbA-L deletion mice (DsbA-L^flox/flox^) were generated by the laboratory of Dr. Feng Liu. Sftpc-CreERT2 mice were provided by Cyagen Biosciences. WT C57BL/6 mice were obtained from Hunan SJA Laboratory Animal Co., Ltd. All mice were housed at a temperature of 22 ± 2 °C, a humidity of 70%, and a 12-h light–dark cycle with free access to a standard rodent diet and water. SftpcCreERT2^+/+^; DsbA-L^flox/flox^ and SftpcCreERT2^±^; DsbA-L-KO mice were administered tamoxifen (50 mg/kg) intraperitoneally for 7 days. WT and DsbA-L-KO mice were anesthetized with 50 mg/kg sodium pentobarbital. Subsequently, mice were administered BLM (5 mg/kg, Nippon Kayaku) in 50 μL of normal saline intratracheally (Ruan et al. 2020), designated as the WT + Model group and the DsbA-L-KO + Model group. Mice in the WT and DsbA-L-KO groups received the same volume of normal saline. All mice were euthanized on day 21 after BLM injection to collect lung tissue. All animal experiments were conducted in accordance with the guidelines approved by the Animal Care Ethics Committee of Second Xiangya Hospital, People’s Republic of China, and ethical approval was obtained. The sequence of mouse identification is presented in Table 1.

**Table 1:** The mouse identification sequence

Gene	Forward (5’–3’)	Reverse (5’–3’)
DsbA-L Mouse identification	GCCATCGATTCCTGGATGGCTTCTGTTAGAG	ACGTACCCGCGGTCAAAGAGTGCCACTCTGCAATG
Sftpc-CreERT2	TGCTTCACAGGGTCGGTAG	ACACCGGCCTTATTCCAAG
Wildtype	TGCTTCACAGGGTCGGTAG	CATTACCTGGGGTAGGACCA

### Cell culture, transfection, and treatment

The MLE-12 cell line, which belongs to mouse alveolar type II epithelial cells (Xiao et al. 2020), was obtained from the Shanghai Zhongqiao Xinzhou Biotechnology Co., Ltd. (ZQ0470) and maintained in a 5% CO_2_ atmosphere at 37℃. The plasmids (oe-NC, sh-NC, oe-DsbA-L, and sh-DsbA-L) were transfected into MLE-12 cells using the lipofectamine 2000 kit (2028090, Invitrogen). MLE-12 cells were then incubated with 10 ng/mL TGF-β1 for 48 h to mimic PF (Liu et al. 2021).

In the indirect co-culture experiment, mouse macrophage cells (RAW264.7 cell line, ZQ0098, Shanghai Zhongqiao Xinzhou Biotechnology Co., Ltd.) were incubated in transwell chambers. The lower chamber was supplemented with MLE-12 cells transfected with plasmids (sh-NC, sh-DsbA-L, oe-NC or oe-DsbA-L) and exposed to TGF-β1. Following a 24-h incubation period, the co-culture system was stimulated with 8 μg/mL AKT1 agonist SC79 (Yang et al. 2020a) or 20 μM NLRP3 agonist nigericin (Zhang et al. 2020).

Another co-culture system was established to investigate the relationship between alveolar epithelial cells and fibroblasts according to the previous procedure (Li et al. 2020). Briefly, MLE-12 cells were transfected with sh-NC or sh-DsbA-L and subsequently treated with or without 10 ng/mL TGF-β1 for 48 h. The treated MLE-12 cells were cultured in serum-free medium for 24 h, and the conditioned medium (CM) was collected. The CM was transferred to mouse lung fibroblasts (LFs; CP-M006, Procell) and cultured for another 24 h.

### Tissue staining

Lung tissues were collected from patients and animals and observed by hematoxylin–eosin (HE) and Masson staining. Lung tissue sections were subjected to sequential staining with hematoxylin (AWI0001, Abiowell) and eosin (AWI0029, Abiowell) to observe the physiological morphological changes. Lung tissue sections were stained in accordance with the instructions provided by the Masson staining kit (AWI0253, Abiowell) to assess the degree of tissue fibrosis.

### Immunohistochemistry (IHC) and Immunocytochemistry (ICC)

The content of relative cytokines in lung tissue and MLE-12 cells was detected using IHC and ICC, respectively. Briefly, lung tissue sections were subjected to hot antigen retrieval and supplemented with 1% periodic acid solution for incubation 10 min to inactivate endogenous enzymes. After fixation, MLE-12 cells were incubated with 3% H_2_O_2_ for 10 min to inactivate endogenous enzymes. The sections were then incubated with primary antibodies, including collagen I (1:100, 14695-1-AP, Proteintech), collagen III (1:100, 13548-1-AP, Proteintech), α-SMA (1:300, 14395-1-AP, Proteintech), fibronectin (1:200, 15613-1-AP, Proteintech), TGF-β1 (1:200, ab215715, Abcam), p-SMAD3 (1:200, ab254407, Abcam), SMAD3 (1:200, ab202445, Abcam), p-AKT1 (1:200, 11962S, CST), AKT1 (1:200, 10176-2-AP, Proteintech), NLRP3 (1:400, 19771-1-AP, Proteintech), and DsbA-L (1:100, 81527-1-RR, Proteintech). The sections were incubated with the secondary antibody HRP goat anti-rabbit IgG (AWS0005, Abiowell) at room temperature for 30 min and DAB working solution (ZLI-9018, Beijing Zhongshan Jinqiao Biotechnology Co., Ltd.). The images were observed and collected using a microscope (BA210T, Motic).

### Western blot

The total protein was extracted from lung tissue and cells using radio-immunoprecipitation assay (RIPA) buffer (AWB0136, Abiowell). The total protein was then separated using SDS-PAGE and transferred to nitrocellulose membranes. Then, the membranes were blocked with 5% skimmed milk and incubated with primary antibodies, including DsbA-L (1:1000, 14535-1-AP, Proteintech), p-AKT1 (1:500, ab38449, Abcam), AKT1 (1:5000, 60203-2-Ig, Proteintech), NLRP3 (1:1000, ab263899, Abcam), fibronectin (1:1000, 15613-1-AP, Proteintech), vimentin (1:5000, 10366-1-AP, Proteintech), E-cadherin (1:5000, 20874-1-AP, Proteintech), collagen I (1:1000, ab270993, Abcam), collagen III (1:1000, 13548-1-AP, Proteintech), TGF-β1 (1:2000, 21898-1-AP, Proteintech), p-SMAD3 (1:2000, ab52903, Abcam), SMAD3 (1:3000, 66516-1-Ig, Proteintech) and β-actin (1:5000, 66009-1-Ig, Proteintech), and secondary antibodies HRP goat anti-mouse IgG (1:5000, SA00001-1, Proteintech) or HRP goat anti-rabbit IgG (1:6000, SA00001-2, Proteintech). The protein bands were visualized using SuperECL Plus (AWB0005, Abiowell) and captured by ChemiScope6100 (CLiNX). The abundance of target proteins was quantified with β-actin as the internal reference.

### Quantitative real-time PCR (qRT-PCR)

Total RNA was isolated from lung tissue and cells using TRIzol (15596026, Thermo). A total of 50 pg-5 µg of total RNA was reverse transcribed to produce cDNA using the HiFiScript cDNA Synthesis Kit (CW2569, CoWin Biosciences). The cDNA of the samples was amplified with the UltraSYBR Mixture (CW2601, CoWin Biosciences) on the QuantStudio 1 Real-Time PCR (Thermo). The relative expression of target genes was calculated with reference to GAPDH. The sequences of the primers are presented in Table 2.

**Table 2:** Primer sequences

Gene	Forward (5’–3’)	Reverse (5’–3’)
H-DsbA-L	AAGTTTGTCTGCCATGCGTT	CCTGGGGAAGGTCAGAAAGT
H-NLRP3	GCCACGCTAATGATCGACT	TCTTCCTGGCATATCACAGT
M-DsbA-L	GTTGCGGCCCACTTTAATCG	ATGTACTGGCCTTTTCGGGG
M-AKT1	CGCCTGCCCTTCTACAACCAG	GCATGATCTCCTTGGCATCCTC
M-NLRP3	CCTCTTTGGCCTTGTAAACCAG	TGGCTTTCACTTCAATCCACT
IL-6	GACTTCCATCCAGTTGCCTT	ATGTGTAATTAAGCCTCCGACT
TNF-α	AGCACAGAAAGCATGATCCG	CACCCCGAAGTTCAGTAGACA
IL-4	ATGTACCAGGAGCCATATCCACGG	TCCCTTCTCCTGTGACCTCGTT
H-GAPDH	ACAGCCTCAAGATCATCAGC	GGTCATGAGTCCTTCCACGAT
M-GAPDH	GCGACTTCAACAGCAACTCCC	CACCCTGTTGCTGTAGCCGTA

### TdT-mediated dUTP nick-end labeling (TUNEL) assay

The degree of apoptosis in MLE-12 cells and lung tissues of rats was validated with a TUNEL Apoptosis Detection kit (40306ES50, YEASEN). In brief, 100 μL of Proteinase K solution was added to each sample at 37 °C for incubation. A total of 50 μL of fresh TdT reaction buffer (comprising 4 μL of TdT enzyme, 45 μL of 5 × equilibration buffer and 1 μL of biotin-11-dUTP) and streptavidin-TRITC solution (comprising 45 μL of labeling buffer and 5 μL of streptavidin-fluorescein) were added successively to MLE-12 cells. Then, 100 μL of 1 × equilibration buffer and 50 μL of fresh TdT reaction buffer (comprising 10 μL of 5 × equilibration buffer, 5 μL of FITC-12-dUTP labeling mix and 1 μL of recombinant TdT enzyme) were added to the lung tissues. Each sample was incubated at 37 °C in the dark. DAPI (AWC0291, Abiowell) was employed to stain the nuclei. The number of positive cells was quantified.

### JC-1 staining and cell apoptosis measurement

The JC-1 kit (C2006, Beyotime Biotechnology) was used to assess the mitochondrial membrane potential of lung epithelial cells in rats. The cells were digested with trypsin (AWC0232, Abiowell) and centrifuged at 1500 rpm for 5 min. The JC-1 solution was then added to each sample for incubation in the dark. The mixture was centrifuged at 4 °C to pellet the cells. The ratio of red and green fluorescence was quantified using flow cytometry (A00-1-1102, Beckman) to assess the degree of cell apoptosis.

### Mitochondrial status

The lung epithelial cells of rats were fixed with 2.5% glutaraldehyde (AWI0097, Abiowell) and 1% osmium tetroxide (18456, TED PELLA). The cells were then dehydrated with ethanol, uranyl acetate (GZ02625, Beijing Zhongjingkeyi Technology), and propylene oxide (M25514, MERYER). Following sectioning and staining, the mitochondrial status was observed under a transmission electron microscope (TEM) (JEM1400, JEOL).

### Flow cytometry

The expression of M2 macrophage markers, CD68 and CD206, was determined by flow cytometry. Briefly, 200 μL of PBS buffer was added to resuspend the cells. A total of 5 μL of CD68 and CD206 antibodies were added to the cells, and the mixture was incubated in the dark for 30min. After washed twice, the cells were resuspended in 200 μL of PBS buffer and subjected to flow cytometry analysis.

### Enzyme-linked immunosorbent assay (ELISA)

The content of TGF-β1 (KE10005, Proteintech) in the cell supernatant was quantified in accordance with the manufacturer’s protocol. Following the addition of the stop solution, the optical density (OD) value was read at 450 nm by a microplate reader (MB-530, HEALES).

### Biochemical tests

According to the instructions of the kit (A030-2-1, Nanjing Jiancheng Bioengineering Institute), the content of hydroxyproline in lung tissues was determined by alkaline hydrolysis method.

### Statistical analysis

All data were presented as the means ± standard deviation. The numerical values were analyzed using GraphPad Prism 8.0 software. Student’s *t-test* was used to compare the difference between two groups. The differences among multiple groups were determined using one-way analysis of variance (ANOVA) and Tukey’s test. *P* < 0.05 was considered to be statistically significant.

## Results

### High expression of DsbA-L in patients with PF

**Fig. 1:**
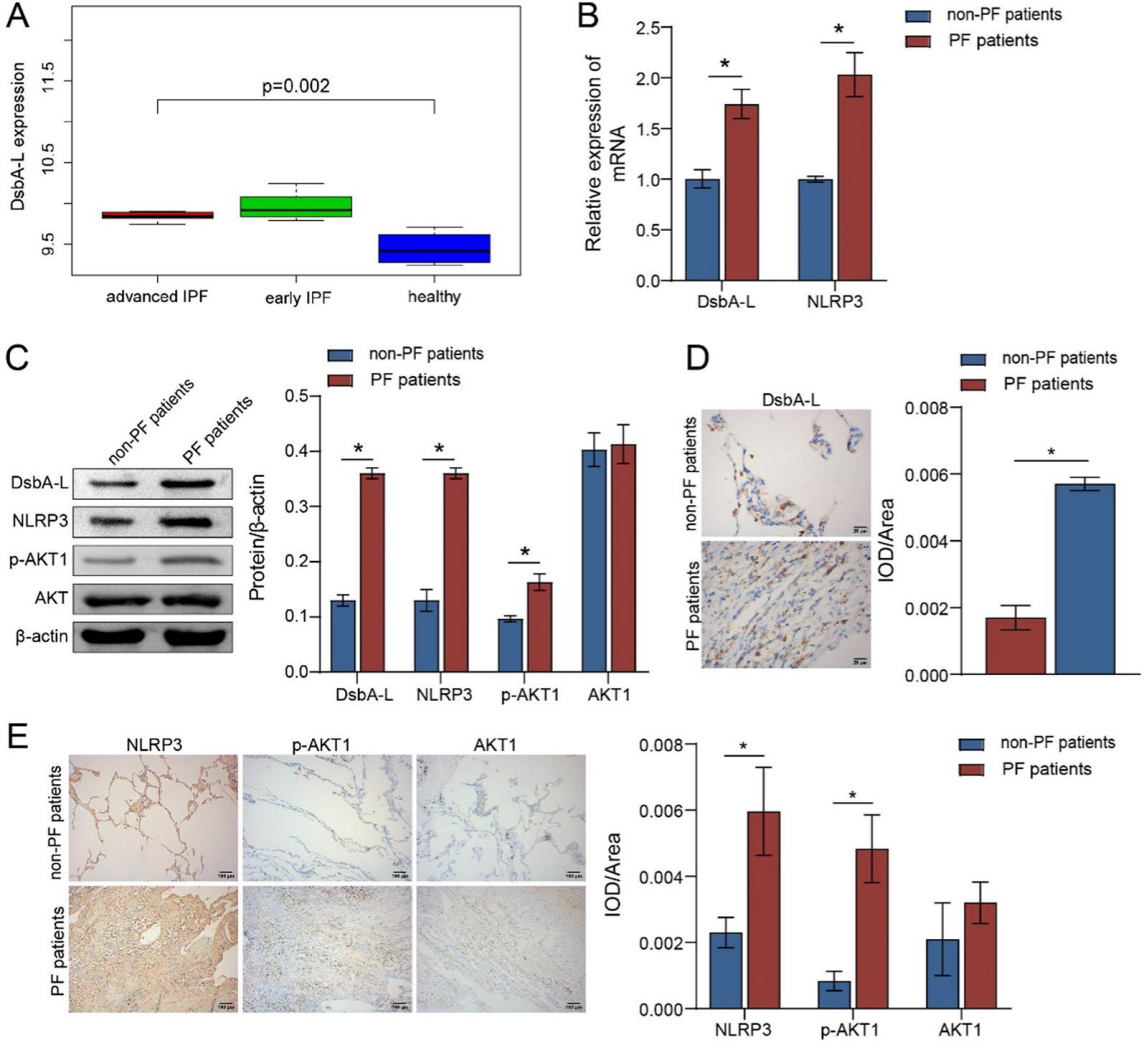
Analysis of histopathological changes in clinical samples with PF. **A** Results from the GEO interstitial lung disease database. **B** Quantitative real-time PCR was used to detect the mRNA levels of *DsbA-L* and *NLRP3*. **C** The protein levels of DsbA-L, p-AKT1, AKT1, and NLRP3 were measured by western blot. **D** Expression levels of DsbA-L were assessed by IHC staining. **E** Expression levels of NLRP3, p-AKT1, and AKT1 were assessed by IHC staining. *P < 0.05

A comparative analysis of the GSE24206 database, comprising lung tissue samples from six healthy controls, eight early IPF patients, and nine advanced IPF patients, revealed that the expression levels of DsbA-L were significantly elevated in both early and advanced IPF patients in comparison to those observed in healthy controls (Fig. 1A). Clinical samples were collected to investigate the accumulation of DsbA-L in patients with PF. The severity of inflammation and tissue fibrosis in the lungs of the clinical samples was assessed by HE and Masson staining. The images in Figure S1A indicated that the alveolar morphology of healthy patients was relatively normal, with little infiltration of inflammatory cells in the pulmonary interstitium and few light blue fibers. The lung structure of patients with PF was damaged, with significant thickening of the alveolar wall and infiltration of inflammatory cells, and a large number of blue collagen deposition in the extracellular matrix (Figure S1A). The staining results indicated that patients with PF exhibited obvious inflammation and fibrosis in lung tissues. Furthermore, the extracellular matrix proteins collagen I, collagen III, α-SMA, and fibronectin were significantly deposited in the model group compared with the control group (Figure S1B-S1D). Subsequently, we sought to determine whether the progression of PF is associated with the production of DsbA-L. The mRNA expression of *DsbA-L* and *NLRP3* in patients with PF was markedly higher than in in non-PF patients (Fig. 1B). Furthermore, the protein abundance of DsbA-L and NLRP3, as well as the ratio of p-AKT1/AKT1, were elevated in patients with PF (Fig. 1C). IHC staining further confirmed the increased activities of DsbA-L, NLRP3, and AKT1 in patients with PF (Fig. 1D, E). Therefore, it can be hypothesized that DsbA-L is involved in the development of PF.

### DsbA-L promotes the development of PF through the TGF-β1 pathway

Alveolar epithelial cell damage is a key initiation factor of PF (Zhang et al. 2024a). Extensive loss of alveolar type II epithelial cells activates fibroblasts in vivo and induces fibrosis (Cohen, et al. 2024). TGF-β1 is a well-documented profibrogenic cytokine in the progression of IPF (Wei et al. 2019). MLE-12 belongs to the alveolar type II epithelial cell line, which can mimic PF-like phenotypes after exposure to TGF-β1 (Liu et al. 2021). The investigation into DsbA-L expression at various time points and concentrations induced by TGF-β1 in MLE-12 cells revealed the most notable upregulation of DsbA-L at a concentration of 10 ng/mL following 48 h of TGF-β1 treatment (Figure S2A and S2B). Therefore, subsequent treatment with 10 ng/mL TGF-β1 for 48 h was selected for MLE-12 cells.

**Fig. 2:**
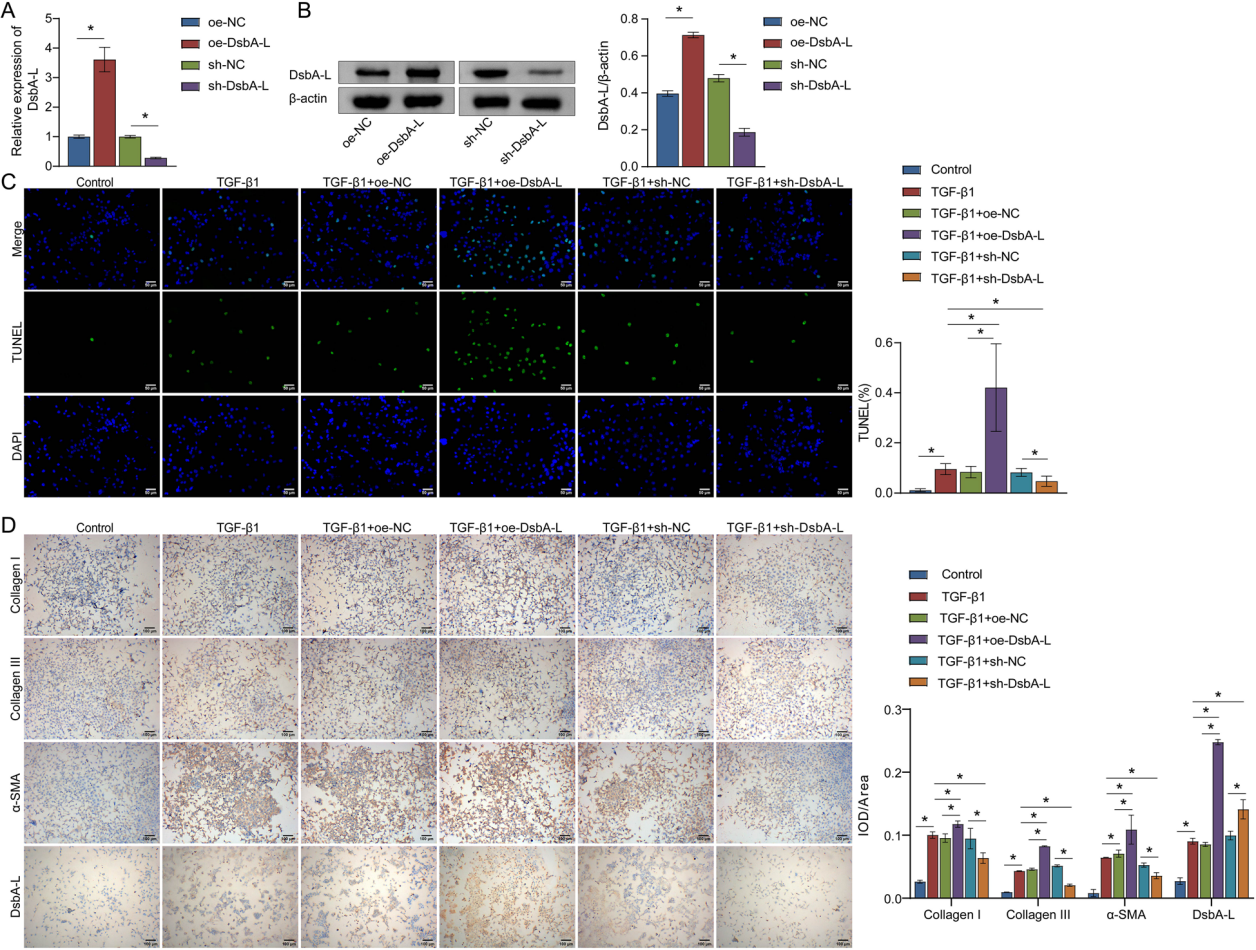

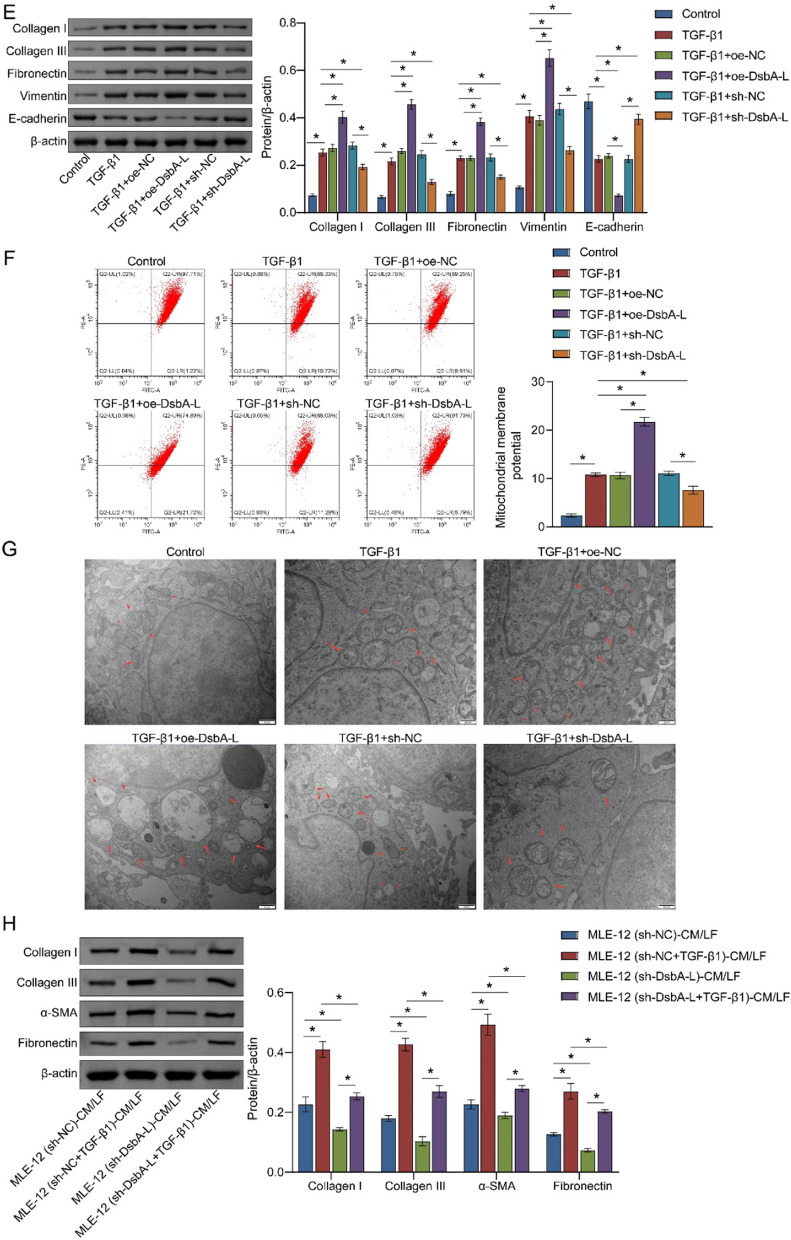
DsbA-L exacerbated the development of PF by activating the TGF-β1 signaling pathway in vitro. **A** The mRNA levels of *DsbA-L* in MLE-12 cells were detected by quantitative real-time PCR. **B** Western blot analysis of DsbA-L. **C** TUNEL-positive cells were counted to assess the degree of apoptosis. **D** Expression of collagen I, collagen III, α-SMA, and DsbA-L based on IHC staining. **E** Western blot analysis of collagen I, collagen III, fibronectin, vimentin, and E-cadherin. **F** Fluorescence in MLE-12 cells with JC-1 was measured by flow cytometry. **G** Transmission electron microscopy (TEM) images of MLE-12 cells with different treatments. Red arrows indicate damaged mitochondria. Scare bar = 500 nm. **H** MLE-12 cells were transfected with sh-NC or sh-DsbA-L with or without TGF-β1 exposure, followed by co-culture with mouse lung fibroblasts (LFs). Western blot analysis of collagen I, collagen III, α-SMA, and fibronectin. *P < 0.05

To further elucidate the potential mechanism of DsbA-L in the development of PF, the oe-DsbA-L and sh-DsbA-L vectors were successfully expressed in MLE-12 cells. This indicated that transfection with oe-DsbA-L promoted the expression of DsbA-L, while transfection with sh-DsbA-L inhibited its expression (Fig. 2A, B). As shown in Fig. 2C, the apoptosis rate of MLE-12 cells was significantly increased after TGF-β1 treatment. In contrast, the TGF-β1 + oe-DsbA-L group exhibited a higher proportion of apoptosis cells than the TGF-β1 and TGF-β1 + oe-NC groups. However, DsbA-L silencing resulted in a reduced apoptosis rate, suggesting that DsbA-L may exacerbate TGF-β1-induced cell apoptosis. ICC staining and western blot showed that TGF-β1 induced the accumulation of type I and III collagen, α-SMA, and DsbA-L. The increase was amplified by oe-DsbA-L and restricted by sh-DsbA-L (Fig. 2D, E). Our data indicated that DsbA-L positively regulated TGF-β1-mediated expression of type I and III collagen and α-SMA.

EMT is a significant contributor to tissue fibrosis. Its cytological mechanism primarily involves a reduction in the content of epithelial properties-related proteins, such as E-cadherin, and an increase in the expression of mesenchymal properties-related proteins, including fibronectin and vimentin (Shu et al. 2019; Sisto et al. 2021). TGF-β1 upregulated fibronectin and vimentin and downregulated E-cadherin. The changes in marker abundance were more pronounced after transfection with oe-DsbA-L, with expression decreasing in the TGF-β1 + sh-DsbA-L group (Fig. 2E), indicating that DsbA-L amplified the trend of TGF-β1-mediated changes in EMT marker levels. DsbA-L is a mitochondrial-localized chaperone, and impaired mitochondrial function has been linked to the development of IPF (Bueno et al. 2020). Therefore, we proceeded to analyze the mitochondrial membrane potential difference and mitochondrial structure. In comparison to the control group, the mitochondrial membrane potential (MMP) difference, which can be utilized for the measurement of mitochondrial viability and function, was found to be more pronounced in cells treated with TGF-β1. Furthermore, the TGF-β1-induced elevation in MMP difference was increased by oe-DsbA-L and decreased by sh-DsbA-L (Fig. 2F). TEM enables intuitive judgment of mitochondrial morphology, which indicates mitochondrial functional damage and pulmonary fibrosis (Zhang et al. 2024b). The images revealed that TGF-β1 treatment resulted in smaller mitochondria, increased membrane density, and decreased number of cristae. Mitochondrial function was exacerbated by oe-DsbA-L and partially restored with sh-DsbA-L (Fig. 2G). Moreover, we established a co-culture system to explore the mechanism by which alveolar epithelial cells drive PF. The expression of α-SMA, fibronectin, collagen I, and collagen III was increased in MLE-12(sh-NC + TGF-β1)-CM/LF compared with MLE-12(sh-NC)-CM/LF. However, the supernatant of MLE-12 cells transfected with sh-DsbA-L reversed the expression of ECM proteins (Fig. 2H). These findings suggested that DsbA-L silencing in alveolar epithelial cells contributed to the alleviation of TGF-β1-induced ECM deposition in mouse lung fibroblasts.

### DsbA-L affects the positive feedback loop of the TGF-β1/SMAD3 pathway by promoting the expression of AKT1 and NLRP3

**Fig. 3:**
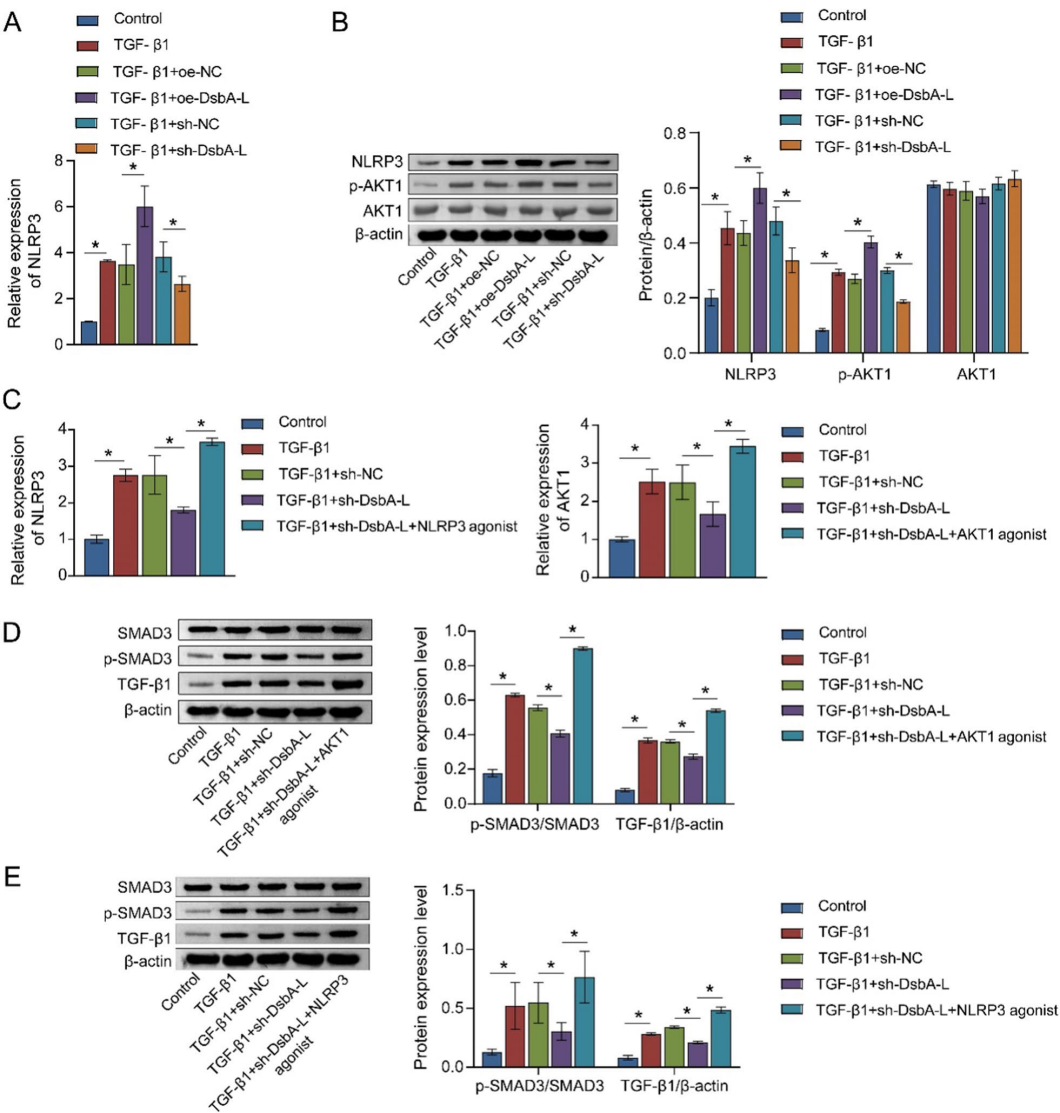
DsbA-L stimulated the TGF-β1/SMAD3 pathway by upregulating AKT1 and NLRP3 expression. **A** The effects of DsbA-L on *NLRP3* mRNA were measured using quantitative real-time PCR. **B** Western blot analysis of NLRP3, p-AKT, and AKT. **C** The activity of AKT1 and NLRP3 in MLE-12 cells was detected by quantitative real-time PCR. Relative protein abundance of SMAD3, p-SMAD3, and TGF-β1 was exhibited in MLE-12 cells after treatment with AKT1 agonist (**D**) or NLRP3 agonist (**E**). *P < 0.05

It has been demonstrated that AKT1 and NLRP3 are involved in the TGF-β1 pathway, which in turn is known to promote PF (Tian et al. 2017; Larson-Casey et al. 2016). We postulated that the effects of DsbA-L on PF may be mediated by AKT1 and NLRP3. The activities of AKT1 and NLRP3 were significantly induced by TGF-β1 in MLE-12 cells. This induction was markedly amplified by oe-DsbA-L and reversed by sh-DsbA-L (Fig. 3A, B). To gain further insight into the roles of AKT1 and NLRP3 in TGF-β1-induced PF, we treated MLE-12 cells with agonists of AKT1 and NLRP3 (Fig. 3C). In comparison to the TGF-β1 + sh-DsbA-L group, the ratio of p-SMAD3/SMAD3 and the abundance of TGF-β1 were found to be higher in the TGF-β1 + sh-DsbA-L + AKT1 agonist and TGF-β1 + sh-DsbA-L + NLRP3 agonist groups (Fig. 3D, E). These findings indicated that DsbA-L facilitated the accumulation of AKT1 and NLRP3, thereby activating the positive feedback loop of the TGF-β1/SMAD3 pathway and affecting the progression of PF.

### MLE-12 cell co-culture induces M2 macrophage polarization in RAW264.7 cells by AKT1 and NLRP3

**Fig. 4:**
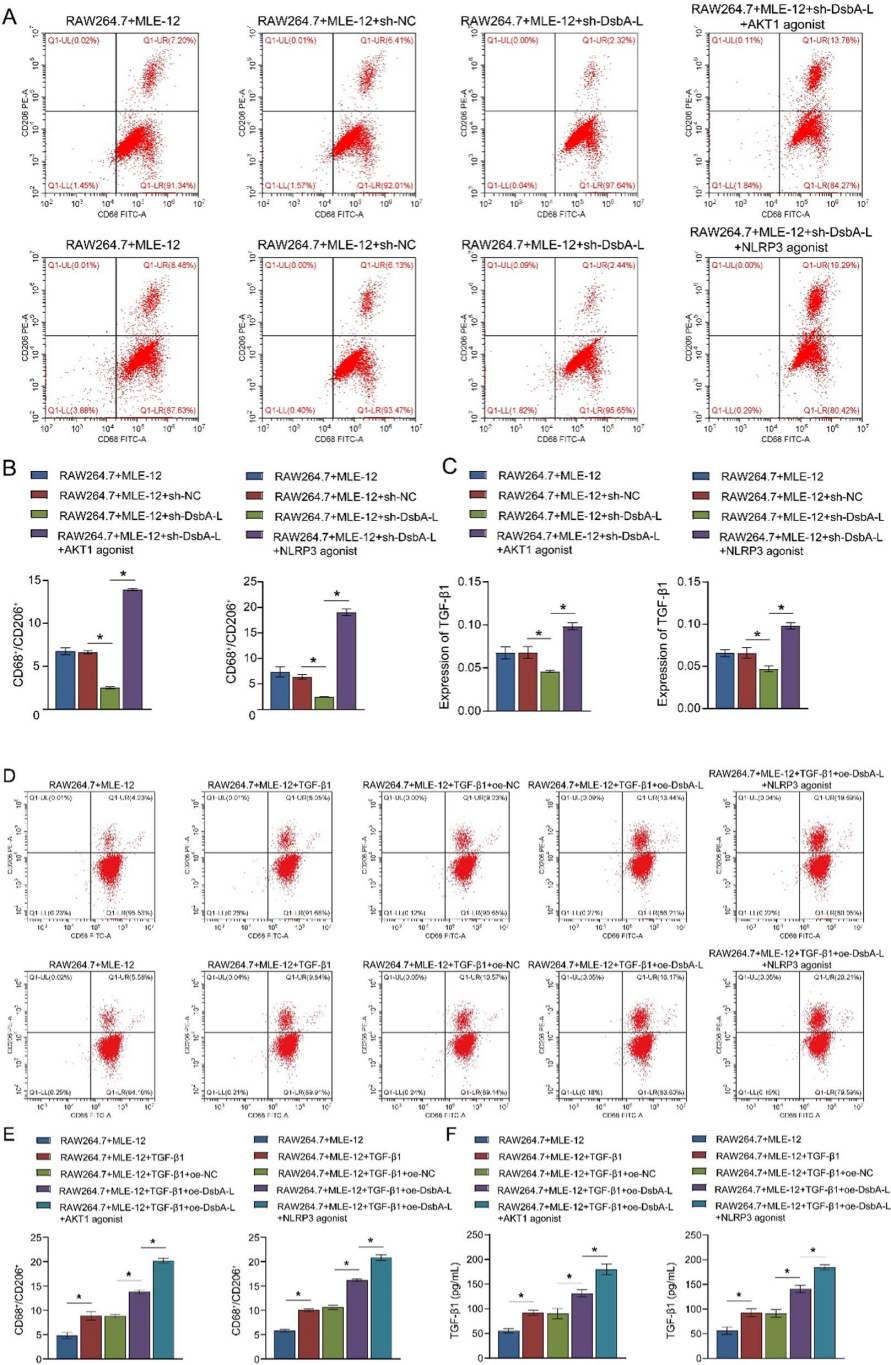
MLE-12 cell co-culture affects M2 macrophage polarization in RAW264.7 cells by AKT1 and NLRP3. The co-culture system of RAW264.7 and MLE-12 cells transfected with sh-NC or sh-DsbA-L was established, followed by exposure to the AKT1 agonist SC79 or the NLRP3 agonist nigericin. **A**, **B** Flow cytometry identification of the M2 macrophage marker CD68^+^/CD206^+^. **C** ELISA was used to measure the expression levels of TGF-β1. The co-culture system of RAW264.7 and MLE-12 cells transfected with oe-NC or oe-DsbA-L was established, followed by exposure to SC79 or nigericin. **D**, **E** Flow cytometry identification of the M2 macrophage marker CD68^+^/CD206^+^. **F** ELISA was used to measure the expression levels of TGF-β1. *P < 0.05

Macrophage M2 polarization is regarded as a pivotal pathological phenotype in the pathogenesis of PF (Wang et al. 2020). The mouse macrophage cell line RAW264.7 cells were co-cultured with TGF-β1-exposed MLE-12 cells without or with prior plasmid transfection. In comparison to the RAW264.7 + MLE-12 + TGF-β1 and RAW264.7 + MLE-12 + TGF-β1 + sh-NC groups, the M2 macrophage marker CD68^+^/CD206^+^ ratio exhibited a decline in cells following the silencing of DsbA-L. Moreover, the administration of AKT1 agonist or NLRP3 agonist led to a significant increase in the CD68^+^/CD206^+^ ratio in comparison to the RAW264.7 + MLE-12 + TGF-β1 + sh-DsbA-L group (Fig. 4A, B). Furthermore, the content of TGF-β1 was found to be negatively regulated by DsbA-L in the co-culture system. However, the administration of AKT1 agonist and NLRP3 agonist reversed the DsbA-L-induced change in the TGF-β1 levels (Fig. 4C). compared to the RAW264.7 + MLE-12 + TGF-β1 and RAW264.7 + MLE-12 + TGF-β1 + oe-NC groups, the CD68^+^/CD206^+^ ratio in cells after DsbA-L overexpression exhibited elevated trends. The increase in CD68^+^/CD206^+^ was further amplified by the administration of AKT1 agonist or NLRP3 agonist compared to the RAW264.7 + MLE-12 + TGF-β1 + oe-DsbA-L group (Fig. 4D, E). Additionally, oe-DsbA-L stimulated an increase in TGF-β1 levels compared to the RAW264.7 + MLE-12 + TGF-β1 + oe-NC group, and further administration of AKT1 agonist and NLRP3 agonist induced this trend (Fig. 4F). Collectively, these findings indicated that DsbA-L contributed to M2 macrophage polarization, which was mediated by AKT1 and NLRP3.

### Establishment of *DsbA-L* knockout mice

To elucidate the role of DsbA-L in the lung, a mouse model was established comprising SftpcCreERT2; DsbA-L-KO mice. Further details regarding the breeding process are provided in Figure S3A. The floxed DsbA-L allele in male mice (DsbA-L^flox/flox^ XY) was crossed with female SftpcCreERT2^+/+^ transgenic mice (SftpcCreERT2^+/+^; DsbA-L^+/+^ XX). Subsequently, heterozygous female offspring (SftpcCreERT2^±^; DsbA-L^flox/+^ XX) were mated with SftpcCreERT2^±^; DsbA-L^flox/flox^ XY males to generate littermate mice. Alternatively, heterozygous female offspring (SftpcCreERT2^±^; DsbA-L^flox/+^ XX) were crossed with heterozygous male offspring (SftpcCreERT2^±^; DsbA-L^flox/+^ XY) to produce littermate mice. The genotypes included SftpcCreERT2^−/−^; DsbA-L-WT, SftpcCreERT2^+/+^; DsbA-L-KO and SftpcCreERT2^±^; DsbA-L-KO. PCR assays confirmed the genotypes, each mouse underwent four sets of PCR. The characteristics of the genotype SftpcCreERT2; DsbA-L-KO mice include amplification of the 1040 bp DNA fragment of the floxed allele, defective amplification of the 915 bp DNA fragment of the wild-type allele, amplification of the Cre gene of the 210 bp DNA fragment, and amplification of the 327 bp DNA fragment of the Cre wild-type allele (Figure S3B). In the absence of tamoxifen induction, DsbA-L expression was not significantly different in C57BL/6 mice, SftpcCreERT2^−/−^; DsbA-L-WT mice, SftpcCreERT2^±^; DsbA-L-KO, and SftpcCreERT2^+/+^; DsbA-L-KO mice (Figure S3C and S3D). However, after intraperitoneal injection of tamoxifen (50 mg/kg) for 7 days, DsbA-L expression was reduced in SftpcCreERT2^±^; DsbA-L-KO and SftpcCreERT2^+/+^; DsbA-L-KO mice (Figure S3E and S3F).

### DsbA-L accelerates the progression of BLM-induced PF in vivo

In the BLM-induced mouse model of PF, lung inflammation and fibrosis were observed to worsen with prolonged intervention (Figure S4A and S4B). In addition, the expression of DsbA-L and fibrotic markers in BLM-intervened mice showed a time-dependent increase (Figure S4C and S4D). Therefore, BLM was injected into the trachea of mice and the animals were sacrificed on day 21.

**Fig. 5:**
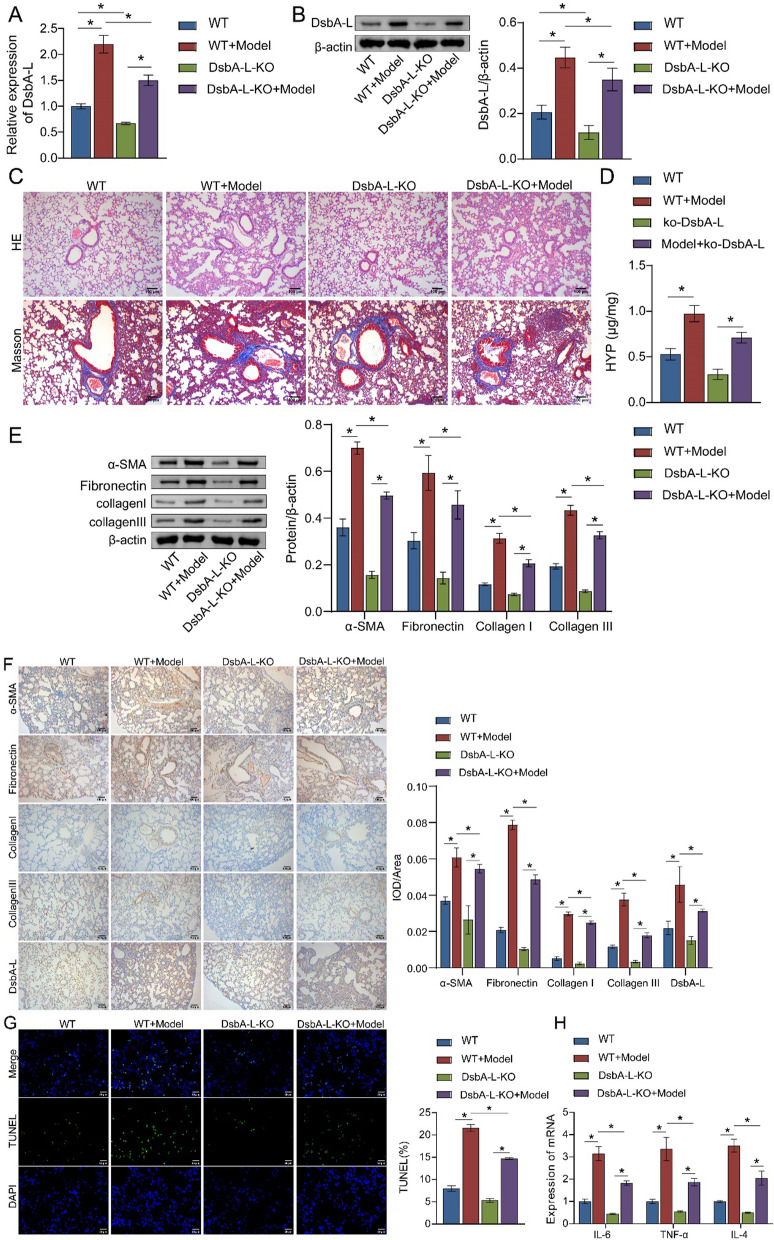
DsbA-L negatively regulates inflammation and fibrosis in BLM-induced mice. The mRNA and protein levels of DsbA-L in lung tissues were determined by **A** quantitative real-time PCR and **B** western blot. **C** Hematoxylin–eosin and Masson staining was performed to observe the structure and inflammation in mouse lung tissues. **D** Hydroxyproline levels. The levels of α-SMA and fibronectin in lung tissues were detected by **E** western blot and **F** immunohistochemistry. **G** Apoptosis was detected by TUNEL assay. **H** Quantitative real-time PCR was used to measure the levels of inflammatory factors *IL-4*, *IL-6*, and *TNF-α* in lung tissues. *P < 0.05

Compared with the WT mouse, BLM induced a significant increase in the expression levels of DsbA-L (Fig. 5A, B), suggesting that the development of PF in mice was accompanied by an increase in DsbA-L levels. According to HE and Masson staining, the lung structure of mice in the model group showed severe damage and massive deposition of ECM, and DsbA-L knockout alleviated inflammation and pulmonary fibrosis in BLM-induced PF-like mice (Fig. 5C). The levels of hydroxyproline were elevated in BLM-induced PF-like mice, whereas DsbA-L knockout reversed this trend (Fig. 5D). Furthermore, the expression levels of α-SMA, fibronectin, collagen I, and collagen III were significantly increased in the lung tissues of PF-like mice, which were significantly suppressed after DsbA-L knockout (Fig. 5E). IHC staining indicated that DsbA-L knockout inhibited the accumulation of α-SMA, fibronectin, collagen I, and collagen III in PF-like mice (Fig. 5F). TUNEL analysis showed that BLM induced apoptosis, while DsbA-L knockout inhibited apoptosis in mouse lung tissue (Fig. 5G). Furthermore, the high mRNA levels of the inflammatory factors *IL-4*, *IL-6*, and *TNF-α* induced by BLM were reversed after DsbA-L knockout (Fig. 5H). These results demonstrated that DsbA-L had an important role in promoting the progression of PF in vivo.

### DsbA-L regulates the TGF-β1/SMAD3 pathway and M2 macrophage polarization in vivo

**Fig. 6:**
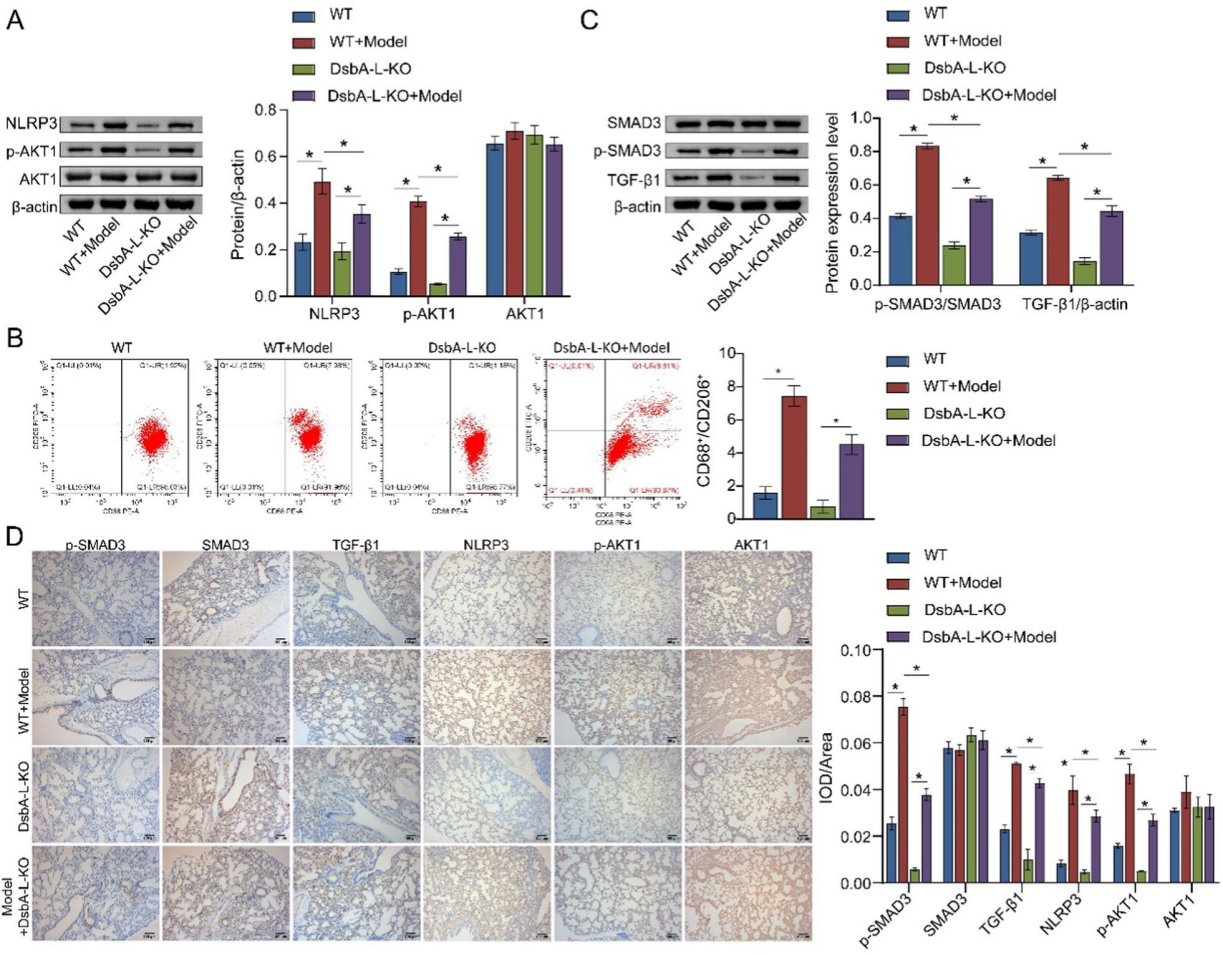
DsbA-L activates AKT1 and NLRP3 through the TGF-β1/SMAD3 pathway and M2 macrophage polarization in vivo. **A** Western blot analysis for p-AKT1, AKT1, and NLRP3. **B** Flow cytometry was used to detect mitochondrial membrane potential with JC-1 staining. **C** Western blot analysis of p-SMAD3, SMAD3, and NLRP3. **D** IHC staining of p-SMAD, SMAD3, TGF-β1, NLRP3, p-AKT1, and AKT1. *P < 0.05

To determine whether DsbA-L is involved in the AKT1- and NLRP3-activated signaling cascade in vivo, we examined the levels of AKT1 and NLRP3 in mouse lung tissue. BLM-induced expression of AKT1 and NLRP3 in the mouse lung tissue was decreased in the DsbA-L-KO + Model group compared with the WT + Model group (Fig. 6A), indicating that DsbA-L positively regulated the abundance of AKT1 and NLRP3 in vivo, which was consistent with the cellular experiments. DsbA-L knockout significantly decreased the BLM-stimulated ratio of CD68^+^/CD206^+^ (Fig. 6B), suggesting that DsbA-L played a critical role in the functional status of BLM-activated M2 macrophages. The ratio of p-SMAD3/SMAD3 and the expression of TGF-β1 were enhanced by BLM treatment, and this trend was inhibited by DsbA-L knockout in mouse lung tissue (Fig. 6C). In addition, the levels of p-SMAD3, TGF-β1, NLRP3, and p-AKT1 were elevated in the WT + Model group, whereas DsbA-L knockout reversed these trends (Fig. 6D). Taken together, the in vivo evidence supported that DsbA-L exacerbated the development of BLM-induced PF in mice by promoting the expression of AKT1 and NLRP3, which in turn activated the TGF-β1/SMAD3 pathway and M2 macrophage polarization.

## Discussion

PF leads to an irreversible decline in lung function and ultimately respiratory failure (Spagnolo et al. 2014). Therefore, there is an urgent need to find effective and safe therapeutic drugs. In the present report, we first confirmed that both patients with PF and BLM-induced PF-like models had higher DsbA-L expression than controls. Based on this observation, we then investigated the function of DsbA-L in the progression of PF. We reported that loss of DsbA-L attenuated the accumulation of AKT1 and NLRP3 to inhibit the TGF-β1/SMAD3 signaling and M2 macrophage polarization, which in turn protected mice from BLM-induced PF. These findings mechanistically explain that DsbA-L may be a promising target for the treatment of PF.

DsbA-L has been found to be widely involved in inflammation, insulin resistance, renal tubular damage, and adiponectin multimerization (Bai et al. 2017; Yang et al. 2019; Zhou et al. 2020; Oniki et al. 2020a). However, reports on the role of DsbA-L in tissue fibrosis remain scarce. Recently, studies have shown that DsbA-L contributes to the progression of renal fibrosis. The expression of DsbA-L was significantly increased in renal fibrosis patients and models, and loss of DsbA-L ameliorated renal fibrosis and tubular apoptosis (Li et al. 2020). In the present study, we reported that DsbA-L, as a distinct target, may contribute to the pathogenesis of PF. Experimental data showed that the high expression of DsbA-L was strongly stimulated in patients with PF and PF-like models. The development of PF is manifested by impaired crosstalk between epithelial cells, the ECM, and nearby mesenchymal and inflammatory cells (Hewlett et al. 2018). We then confirmed that DsbA-L promoted the accumulation of extracellular matrix markers collagen I and III and mesenchymal markers α-SMA and fibronectin, and accelerated lung epithelial cell apoptosis and inflammatory responses. Taken together, these data support a role for DsbA-L in mediating PF.

In the lesion process of PF, the profibrotic mediator TGF-β1 accelerates downstream gene transcription to promote collagen and fibronectin production, thereby increasing the deposition of ECM (Inui et al. 2021). TGF-β1 activates the classical SMAD-dependent signaling cascade, and the phosphorylated SMAD protein transduces the signal downstream to regulate the transcription of target genes (Tzavlaki and Moustakas 2020). We further confirmed that DsbA-L exacerbated the pathological phenotype of PF through TGF-β1/SMAD3 signaling. DsbA-L contributed to the expression of TGF-β1 and p-SMAD3, which is consistent with previous reports (Yang et al. 2020b; Tong et al. 2021). TGF-β1-mediated extracellular matrix deposition and lung epithelial cell apoptosis were accelerated by DsbA-L production. We also found that TGF-β1 induced DsbA-L upregulation. Lu et al*.* showed that MBNL2 overexpression caused increased expression of MBNL2 and TGF-β1 in cardiac fibroblasts, and further application of a TGF-β1 receptor inhibitor (SB431542) reduced MBNL2 and TGF-β1 expression (Lu, et al. 2024). Li et al. proposed that TGF-β1 expression in LX-2 cells was limited after the knockdown of EXO1, and supplementation of TGF-β1 upregulated EXO1 expression in LX-2 cells (Li et al. 2023). These may suggest that DsbA-L affects pulmonary fibrosis by regulating the positive feedback loop of the TGF-β1/SMAD3 pathway. Previous studies have suggested that inhibition of M2 macrophage polarization is beneficial to protect against BLM-induced PF in mice (Gharib et al. 2014). The production and maintenance of TGF-β1 is also dependent on the fibrotic microenvironment generated by M2 macrophages (Yu et al. 2017). As a result, the enhanced macrophage infiltration by DsbA-L may be one of the reasons for the increased accumulation of TGF-β1 in BLM-induced mice. In addition, IL-4 is a potent inducer of M2 macrophages (Zhu et al. 2017). We reported that DsbA-L knockout caused a reduction in IL-4 levels, which in turn inhibited M2 macrophage polarization. Taken together, these results suggested that DsbA-L activated TGF-β1/SMAD3 signaling and increased the levels of TGF-β1 and IL-4 to induce M2 macrophage polarization and ultimately mediate the progression of PF.

AKT is a major regulator of cell survival and is also involved in the proliferation and activation of myofibroblasts in the lungs (Wang et al. 2020). Studies have shown that AKT attenuates the development of PF by negatively regulating macrophage apoptosis and the release of profibrotic cytokines (Nie et al. 2019; Larson-Casey et al. 2016). The progression of PF is accompanied by an inflammatory response that relies on the activation of innate immune receptors, such as inflammasomes (Zhang et al. 2021). The NLRP3 inflammasome contributes to the conversion to EMT and accelerates fibrosis formation (Lv et al. 2018). It has been shown that NLRP3 induces increased secretion of IL-4 and leads to polarization of macrophages towards M2 macrophages (Liu et al. 2018). In the present study, we found that AKT1 and NLRP3 induced the accumulation of TGF-β1, which is consistent with previous reports (Jiang et al. 2021; Vaz de Paula et al. 2021). DsbA-L significantly induced the activation of AKT1 and NLRP3 in PF-like models. The AKT1 agonist and the NLRP3 agonist reversed the inhibitory effect of DsbA-L silencing on the TGF-β1/SMAD3 pathway and M2 macrophage polarization in co-cultured cells. They also exacerbated the effects of DsbA-L overexpression on co-cultured cells. These data indicated that DsbA-L contributed to the promotion of TGF-β1/SMAD3 signaling and macrophage M2 polarization through the activation of AKT1 and NLRP3, thereby accelerating the development of PF.

However, our study has limitations. DsbA-L, a potential antioxidant stress agent, plays an important role in the regulation of respiratory function in the elderly (Oniki et al. 2020b). Oxidative stress leads to the production of reactive oxygen species, which activates NLRP3 to be involved in the pathogenesis of PF (Min et al. 2022). Therefore, it will be of great interest in future studies to elucidate the potential role of DsbA-L in inflammation and oxidative stress-induced PF. More examination data are needed to support the lung function of *DsbA-L* knockout mice. The delicate role of DsbA-L in fibroblasts, macrophages, and other lung cells in PF will be explored in future studies. In addition, *DsbA-L* rs1917760 polymorphism may affect the progression of PF (Oniki et al. 2020b). The impact of *DsbA-L* gene polymorphism on gene expression and activity in the lung will be explored in future studies. More evidence is needed to support the role of AKT and NLRP3 in DsbA-L-mediated PF progression.

In conclusion, our study reports a novel target of DsbA-L and demonstrates that DsbA-L could exacerbate BLM-induced PF through the regulation of TGF-β1 and/SMAD3 signaling and macrophage polarization, providing clues for exploring potential drugs for the treatment of PF.

## Supplementary Information

Supplementary material 1.

Supplementary material 2.

Supplementary material 3.

Supplementary material 4.

## Data Availability

No datasets were generated or analysed during the current study.
