# Supplementary figures and images for: DsbA-L activates TGF-β1/SMAD3 signaling and M2 macrophage polarization by stimulating AKT1 and NLRP3 to promote pulmonary fibrosis
Source: Mol Med. 2024 Nov 23;30:228. doi: 10.1186/s10020-024-00983-9 (PMC11585156; doi:10.1186/s10020-024-00983-9)

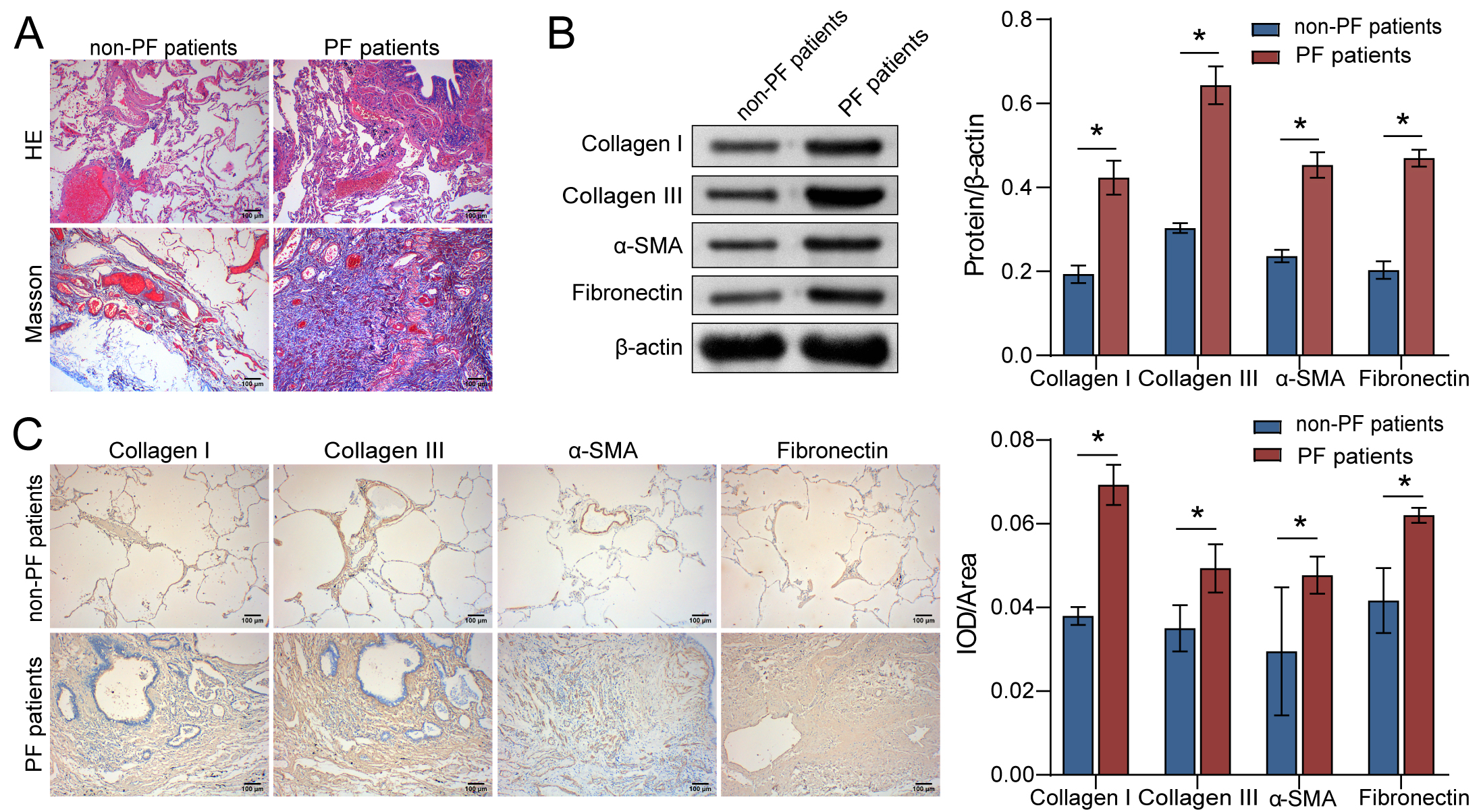

Supplement: Supplementary file 1 — Supplementary material 1. [file 10020_2024_983_MOESM1_ESM.jpg]

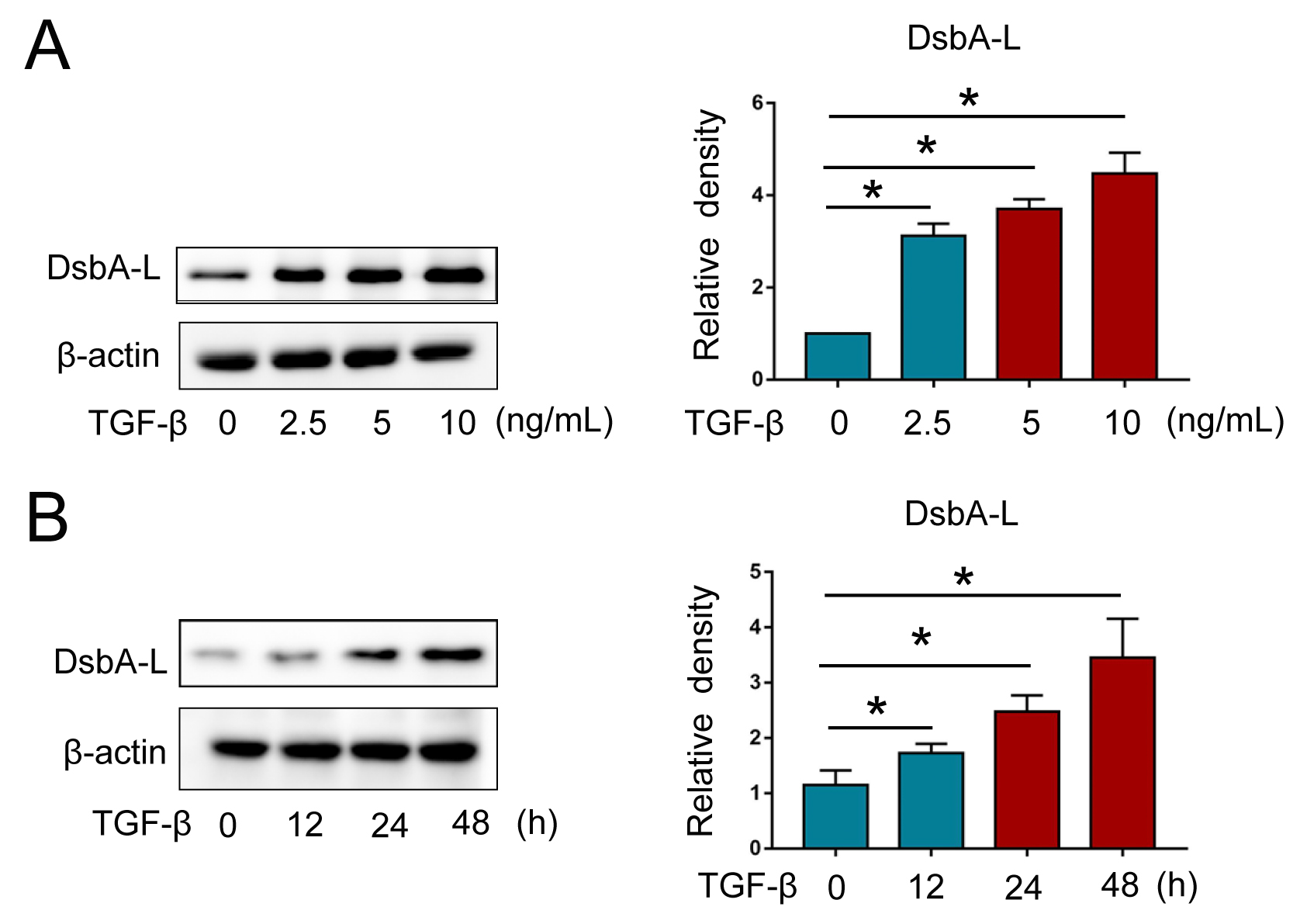

Supplement: Supplementary file 2 — Supplementary material 2. [file 10020_2024_983_MOESM2_ESM.jpg]

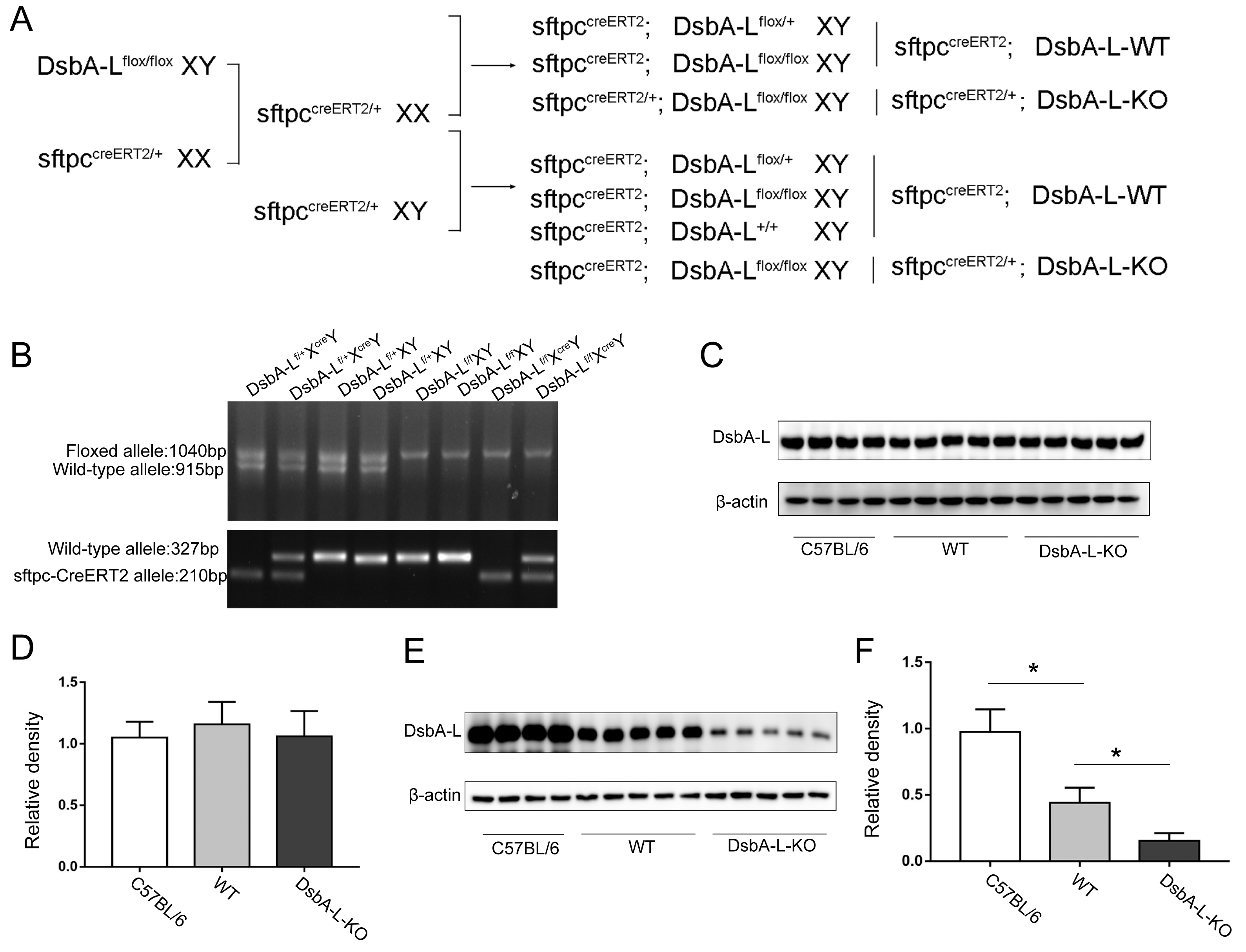

Supplement: Supplementary file 3 — Supplementary material 3. [file 10020_2024_983_MOESM3_ESM.jpg]

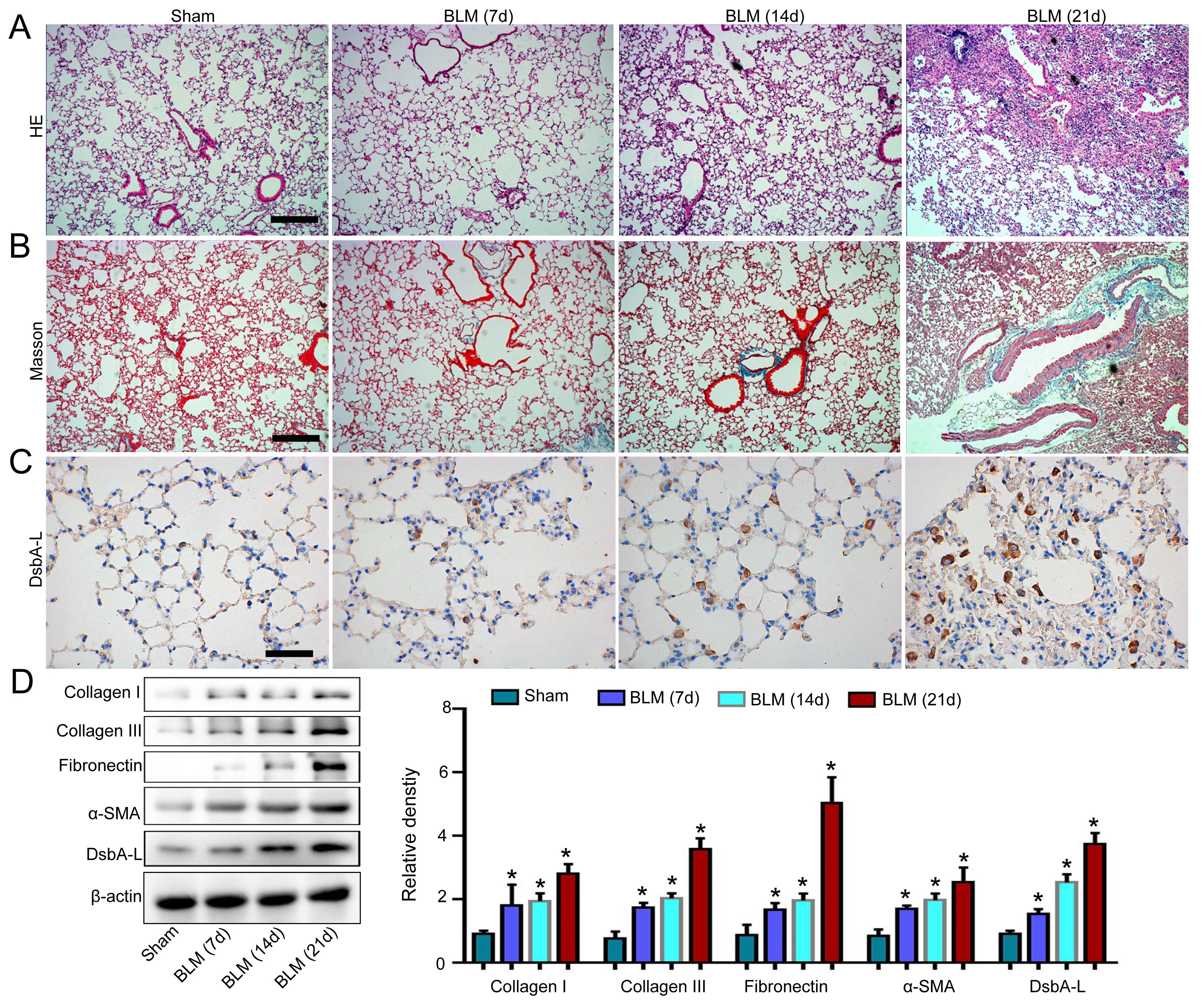

Supplement: Supplementary file 4 — Supplementary material 4. [file 10020_2024_983_MOESM4_ESM.jpg]
